# Neuroprotective Effect of the Combination of Citicoline and CoQ10 in a Mouse Model of Ocular Hypertension

**DOI:** 10.3390/antiox14010004

**Published:** 2024-12-24

**Authors:** José A. Matamoros, Sara Rubio-Casado, José A. Fernández-Albarral, Miguel A. Martínez-López, Elena Salobrar-García, Eva M. Marco, Victor Paleo-García, Rosa de Hoz, Inés López-Cuenca, Lorena Elvira-Hurtado, Lidia Sánchez-Puebla, José M. Ramírez, Juan J. Salazar, Meritxell López-Gallardo, Ana I. Ramírez

**Affiliations:** 1 Ramon Castroviejo Institute for Ophthalmic Research, Complutense University of Madrid, 28040 Madrid, Spain; jomatamo@ucm.es (J.A.M.); srubio02@ucm.es (S.R.-C.); joseaf08@ucm.es (J.A.F.-A.); miguma19@ucm.es (M.A.M.-L.); elenasalobrar@med.ucm.es (E.S.-G.); emmarco@ucm.es (E.M.M.); rdehoz@med.ucm.es (R.d.H.); inelopez@ucm.es (I.L.-C.); marelvir@ucm.es (L.E.-H.); lidsan02@ucm.es (L.S.-P.); ramirezs@med.ucm.es (J.M.R.); jjsalazar@med.ucm.es (J.J.S.); 2 Health Research Institute of the Hospital Clínico San Carlos (IdISSC), 28040 Madrid, Spain; 3 Department of Immunology, Ophthalmology and ENT, Faculty of Optics and Optometry, Complutense University of Madrid, 28040 Madrid, Spain; 4 Department of Immunology, Ophthalmology and ORL, Faculty of Medicine, Complutense University of Madrid, 28040 Madrid, Spain; 5 Department of Genetics, Microbiology and Physiology, Faculty of Biological Sciences, Complutense University of Madrid, 28040 Madrid, Spain; 6 Department of Physiology, Faculty of Medicine, Complutense University of Madrid, 28040 Madrid, Spain; vipaleo@ucm.es

**Keywords:** ganglion cells, retina, visual pathway, experimental glaucoma, citicoline, coenzyme Q10, Brn3a, melanopsin, neuroprotection

## Abstract

Glaucoma is a neurodegenerative disease characterized by the loss of retinal ganglion cells (RGCs), with intraocular pressure (IOP) being its primary risk factor. Despite controlling IOP, the neurodegenerative process often continues. Therefore, substances with neuroprotective, antioxidant, and anti-inflammatory properties could protect against RGC death. This study investigated the neuroprotective effects on RGCs and visual pathway neurons of a compound consisting of citicoline and coenzyme Q10 (CoQ10) in a mouse model of unilateral, laser-induced ocular hypertension (OHT). Four groups of mice were used: vehicle group (*n* = 6), citicoline + CoQ10 group (*n* = 6), laser–vehicle group (*n* = 6), and laser–citicoline + CoQ10 group (*n* = 6). The citicoline + CoQ10 was administered orally once a day starting 15 days before laser treatment, continuing until sacrifice (7 days post-laser). Retinas, the dorsolateral geniculate nucleus (dLGN), the superior colliculus (SC), and the visual cortex (V1) were analyzed. The citicoline + CoQ10 compound used in the laser–citicoline + CoQ10 group demonstrated (1) an ocular hypotensive effect only at 24 h post-laser; (2) prevention of Brn3a+ RGC death in OHT eyes; and (3) no changes in NeuN+ neurons in the dLGN. This study demonstrates that the oral administration of the citicoline + CoQ10 combination may exert a neuroprotective effect against RGC death in an established rodent model of OHT.

## 1. Introduction

Glaucoma is a multifactorial neurodegenerative disease characterized by the progressive death of retinal ganglion cells (RGCs), ultimately leading to irreversible blindness [1].

RGCs can be functionally divided into two subtypes: those involved in image formation, which express Brn3a; and intrinsically photosensitive retinal ganglion cells (ipRGCs), which are primarily involved in the circadian photoentrainment, pupillary reflexes, and the regulation of pineal melatonin secretion [2]. The ipRGCs account for approximately 2–3% of all RGCs [2]. There are six subtypes of ipRGCs (M1–M6); however, only the M1–M3 subtypes express immunodetectable levels of melanopsin [3]. There is evidence that ipRGCs may also be affected in glaucoma, potentially leading to systemic issues related to their dysfunction [3,4]. However, various studies suggest that ipRGCs may be less vulnerable to degeneration compared to other RGCs in rodent models of glaucoma [2,4]. As for human studies, the data are limited, but some reports indicate that ipRGCs are more resistant to degeneration in severe glaucoma, with only a 50% loss compared to the near-complete loss of other RGCs [5].

It is also well established that neurodegenerative processes in glaucoma are not limited to the retina and optic nerve but are associated with alterations in higher visual centers [6,7,8,9]. In humans, all layers of the lateral geniculate nucleus (LGN), where 90% of RGCs project, are affected [10,11]. Furthermore, patients with glaucoma exhibit significant loss of function in the primary visual cortex (V1), as demonstrated by functional magnetic resonance imaging (fMRI) [12] and visual evoked potentials (VEPs) [7]. This disruption of the visual pathway has also been observed in rodent models of ocular hypertension, where neuronal morphological changes have been noted in both the LGN and superior colliculus (SC), the latter being the projection target of 90% of RGCs [6].

Elevated intraocular pressure (IOP) is the primary risk factor for glaucoma; however, in many cases, despite well-controlled IOP, RGC death continues, indicating the progression of the neurodegenerative process [13,14]. This suggests the involvement of additional mechanisms, beyond elevated IOP, in the neurodegeneration process. Noteworthy among these mechanisms are vascular dysregulation, neuroinflammation, oxidative stress, mitochondrial dysfunction, and glutamate neurotoxicity [15,16,17].

Vascular dysregulation from elevated IOP or other vascular risk factors can reduce ocular blood flow [18], causing ischemia–reperfusion events and oxidative stress (OS), which damages cellular components and leads to RGC death [19]. RGCs are highly susceptible to mitochondrial damage due to their high energy demands [20]. OS can impair mitochondria, reduce ATP production, and increase ROS, leading to cell death [21].

Neuroinflammation also plays a role, as glial cell activation from apoptotic signals and elevated IOP releases pro-inflammatory factors, further inducing RGC death [22,23,24].

Additionally, increased extracellular glutamate from dying RGCs and impaired reuptake by astrocytes overstimulates NMDA receptors, causing calcium influx and RGC death [25].

Currently, there are compounds that can aid in neuroprotection when damage occurs in the CNS, among which citicoline and coenzyme Q10 (CoQ10) are notable. It has been observed that citicoline and CoQ10 could exert numerous neuroprotective functions in the CNS, potentially helping to control the damage that occurs in glaucoma.

Citicoline supplementation can stimulate the synthesis of phospholipids, promoting neuronal repair [26,27,28,29]. Additionally, it can increase the viability of vascular endothelial cells, improving ocular blood flow [30,31,32]. Citicoline supplementation can also stimulate antioxidant synthesis, reducing OS [29,32,33]; improve mitochondrial function [26,32,34,35]; and can reduce neuroinflammation [27,28,36] and excitotoxic damage [37,38]. On the other hand, CoQ10 can protect mitochondria from oxidative damage [32,39,40], act as a potent antioxidant [39,41,42,43,44], inhibit glutamate toxicity [32,45,46,47], and block glial activation [29,40,43,47,48].

Based on the beneficial effects that both compounds exert on the CNS, in this study we aimed to analyze whether the combination of citicoline and CoQ10, could prevent the death of RGCs, ipRGCs, and neuronal damage in the visual pathway in a model of laser-induced unilateral hypertension.

## 2. Materials and Methods

### 2.1. Animals

In this study, 24 male CD-1 Swiss albino mice, aged between 12 and 16 weeks and weighing 35–45 g, were used. The animals were obtained from the Charles River Laboratory (Barcelona, Spain) and housed in the animal facility of the School of Medicine at the Complutense University of Madrid (Spain). The animals were maintained under controlled light and temperature conditions (12 h light/dark cycles and 9–24 lux) and had ad libitum access to food (standard diet) and water. The experiments were conducted in accordance with ethical guidelines endorsed by Spanish legislation and the Guidelines for Humane Endpoints for Animals Used in Biomedical Research. The study was approved by the Animal Research Ethics Committee of the Complutense University (Madrid, Spain) and the General Directorate of Agriculture and Food of the Ministry of Economy and Employment of the Community of Madrid (PROEX 091.2/22). All experimental procedures involving animals adhered to institutional guidelines, European Union regulations for the use of animals in research, and the Association for Research in Vision and Ophthalmology (ARVO) Statement for the Use of Animals in Ophthalmic and Vision Research.

### 2.2. Experimental Groups

**Figure 1:**
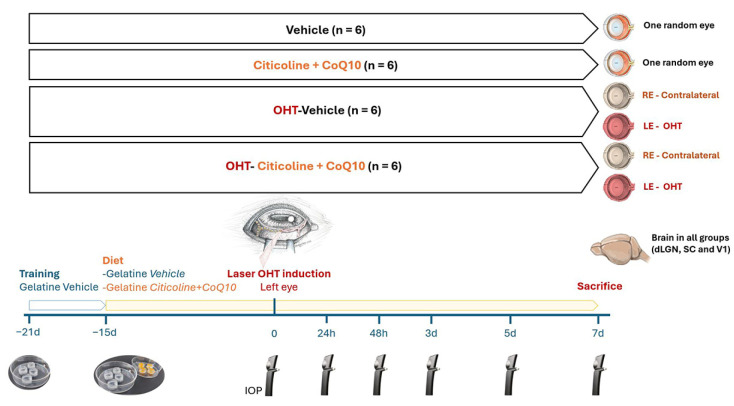
Scheme of the experimental groups of this study.

The animals were divided into 4 experimental groups: (1) vehicle group (*n* = 6), mice that received neutral gelatin throughout the study period and underwent no procedures; (2) citicoline + CoQ10 group (*n* = 6), mice that received citicoline and CoQ10 throughout the study period and underwent no procedures; (3) laser OHT–vehicle group (*n* = 6), mice that received neutral gelatin throughout the study period and were subjected to induced ocular hypertension (OHT); and (4) laser OHT–citicoline + CoQ10 group (*n* = 6), mice that received citicoline and CoQ10 throughout the study period and were subjected to induced OHT. In both the laser OHT–vehicle and laser OHT–citicoline + CoQ10 groups, hypertensive eyes (left eye, OHT) and their contralateral counterparts (right eye, contralateral) were studied at 7 days after laser-induced OHT. For the visual pathway study, in all experimental groups, the dorsolateral geniculate nucleus (dLGN), the superior colliculus (SC). and V1, from the right (dLGN right, SC right, V1 right) and left (dLGN left, SC left, V1left) hemispheres were also analyzed at 7 days after OHT induction (Figure 1).

### 2.3. Citicoline and CoQ10 Treatment

The combined administration of citicoline and CoQ10 was delivered orally via 1 gelatine/day of 0.5 mL, prepared in our laboratory using commercial gelatin (gelatin from porcine skin, Sigma-Aldrich G2500-500G, St. Louis, MO, USA) and potable water. For the animals receiving treatment, the gelatin contained citicoline (Neurotidine^®^, Omikron Pharmaceutical España S.L.U., Barcelona, Spain) (500 mg/kg) and CoQ10 (COQUN^®^ OS, VISUfarma B.V., Madrid, Spain) (200 mg/kg). The combination (citicoline + CoQ10) is commercialized as COQUN Combo (COQUN^®^ combo, VISUfarma S.p.A., Rome, Italy). The daily doses of both compounds were calculated based on previous studies [28,43,47,49], with an average animal weight of 40 g as a reference. The animals in the control group were administered only gelatin as a vehicle. The administration of gelatin (with or without the compound) began 15 days prior to the induction of OHT and continued until the designated sacrifice points (7 days post-OHT induction). This administration period was selected based on the study by Fernández-Albarral et al., 2019 [50]. To ensure the compound was fully ingested by the animals, the person responsible for administering the gelatin monitored them until it was completely consumed.

Before starting the gelatin administration with either the compound or the vehicle, all experimental groups were trained for 1 week with 1 gelatine/day of 0.5 mL (composed of potable water and commercial gelatin, gelatin from porcine skin, Sigma-Aldrich G2500-500G) to familiarize the animals with the ingestion process, ensuring that the mice would consume the full gelatin dose during the experimental phase (Figure 1).

### 2.4. Anesthesia

The surgical procedures (OHT induction and animal sacrifice) were performed under general anesthesia via intraperitoneal injection of ketamine (Anestekin^®^ 100 mg/mL, Dechra Veterinary Products SLU, Barcelona, Spain), medetomidine (Dormisan^®^ 1 mg/mL, Fatro Ibérica, Barcelona, Spain), and saline solution. To aid in the recovery of the animals post-anesthesia, 0.1 mL of atipamezole hydrochloride (Nosedorm^®^ 5 mg/mL, Laboratorios Karizoo S.A., Barcelona, Spain) was administered subcutaneously. Additionally, for OHT induction, a local anesthetic (COLICURSÍ™ ANESTÉSICO DOBLE, 1 mg/mL tetracaine hydrochloride and 4 mg/mL oxybuprocaine hydrochloride, Alcon España, Barcelona, Spain) was applied on the cornea. IOP measurements were performed under inhalational anesthesia with 2% isoflurane in oxygen (Isoflutek^®^ 1000 mg/g, Laboratorios Karizoo S.A., Barcelona, Spain).

### 2.5. Induction of Ocular Hypertension and IOP Measurement

OHT was induced in the left eyes of anesthetized mice using diode laser photocoagulation (Viridis Ophthalmic Photocoagulator—532 nm, Quantel Medical, Clermont-Ferrand, France) on the limbal and episcleral veins, following the protocol established by Ramirez et al., 2023 [51]. The parameters used were a spot size of 50 µm, power of 0.3 W, and duration of 0.5 s. The animals received an average of 80–150 laser impacts, which were applied without any lens. After OHT induction, a drop of Tobradex^®^ (1 mg/mL dexamethasone and 3 mg/mL tobramycin, Alcon, Barcelona) was instilled to prevent corneal dryness, inflammation, and infection.

IOP was measured as previously described [24,52], using a rebound tonometer (TonoLab, Tiolat, OY, Helsinki, Finland), in anesthetized mice (according to the previously mentioned anesthesia protocol). The IOP was recorded in both eyes of mice across all experimental groups. Each IOP reading was the average of 3 independent measurements, with each measurement representing the automatic mean of 6 consecutive readings. Baseline IOP was recorded at the start of the experiment. Following the induction of OHT, IOP was measured at 5 additional time points: 24 h, 48 h, 3, 5, and 7 days after OHT-induction. All IOP measurements were taken at the same time each day (10:00 AM) to minimize variability due to circadian rhythms (Figure 1).

### 2.6. Immunohistochemistry

The animals were euthanized using an overdose of the general anesthesia described in the anesthesia section. The mice underwent transcardiac perfusion, first with a saline solution (0.9% NaCl) followed by a 4% paraformaldehyde (PFA) solution in 0.1 M phosphate buffer. For both the saline and PFA, a volume of 1500 mL/g of body weight was used, and both solutions were maintained at 4 °C. After perfusion was completed, a suture was placed in the upper eyelid to ensure the spatial orientation of the retina. The eyes and brains were then extracted and immersed in 4% PFA at 4 °C for 24 h. In the eyes, the corneas and lenses were removed, and the retinas were subsequently isolated to prepare retinal whole mounts. The retinas and brains were cryoprotected by immersion in sucrose solutions of progressively increasing concentrations (10%, 20%, and 30%) for 1 h, 2 h, and overnight, respectively. Subsequently, the tissues were frozen using liquid nitrogen and stored at −80 °C until further use [53]. After fixation, the brains were first cleaned three times in 0.1 M PBS and then, for cryoprotection, they were rinsed for 48 h at 4 °C in sucrose 11% in 0.1 M PBS and then in sucrose 33% in 0.1 M PBS. The brains were then placed in a freezing environment and kept in Tissue Freezing Medium (Tissue-Tek O.C.T., Sakura Finetek, Torrance, CA, USA) and stored at −20 °C until they were used [54].

#### 2.6.1. Immunohistochemistry in Retinal Tissue

The retinas were immunostained according to previously used protocols [50]. Briefly, double immunostaining was performed to determine the number of surviving RGCs using Brn3a (anti-Brn3a mouse, Millipore, MAB1585, 1:800, Burlington, MA, USA) and the number of ipRGCs that express melanopsin using anti-melanopsin rabbit (Advanced Target Systems, AB-N39, 1:1000), along with their respective secondary antibodies: A488 anti-mouse goat (Invitrogen, A21121, 1:150, Waltham, MA, USA) and A594 anti-rabbit donkey (Invitrogen, A21207, 1:800).

To verify the specificity of the secondary antibodies’ interaction with their respective primary antibodies, three negative controls were implemented. In the first control, the primary antibody was omitted. In the second control, the secondary antibody was omitted. In the third control, both primary and secondary antibodies were excluded, and tissue samples were incubated exclusively in the corresponding diluent solutions. This approach also facilitated the evaluation of endogenous fluorescence levels contributing to the observed signal [50].

Retinal samples were analyzed and photographed using an ApoTome 2 module (Carl Zeiss, Oberkochen, Germany) in conjunction with an Axio CAM 503 Mono high-resolution digital camera (Carl Zeiss) mounted on a Zeiss Axio Imager M.2 fluorescence microscope (Carl Zeiss), as previously reported [55]. The microscope was equipped with Zeiss filter set 64 for Alexa Fluor 594 and Zeiss filter set 10 for Alexa Fluor 488. The ApoTome system facilitates optical sectioning of specimens in conventional microscopy, thereby enhancing contrast and resolution along the optical axis. Retinal whole mounts were scanned across the x-, y-, and z-axes using a motorized stage. Cellular components located in the same x–z plane were considered to lie within the same focal plane. Z-stacks were analyzed with the ZEN2 software version 3.6 (Carl Zeiss, Oberkochen, Germany).

#### 2.6.2. Immunohistochemistry in Visual Pathway Nuclei

The brains were frozen sectioned in 20 μm thick coronal serial sections by using a cryostat (Leica, Wetzlar, Germany, CM3050). The study focused on three specific brain regions of the visual pathway: the dLGN, which, according to the Allen Brain Atlas (Available from “https://mouse.brain-map.org (accessed on 30 June 2024)”), is located between Bregma −1.855 mm and −2.88 mm; the SC, which is located between Bregma −3.08 mm and −3.98 mm; and the V1, which is located between Bregma −2.78 mm and −3.78 mm. Three slices were placed on gelatine-coated slides, which were then left to air-dry for half an hour before being kept in storage at −30 °C until needed.

A total of 5 animals per experimental group and per sacrifice day were randomly selected for immunohistochemical analyses. The brains were immunostained according to previously used protocols [54]. Briefly, immunohistochemistry buffer (IB) was used for washes and incubations. IB contains 0.5% Animal-Free Blocker^®^ and Diluent, R.T.U. (Vector Laboratories, Inc., Newark, CA, USA, Ref. SP-5035), and 0.3% Triton X-100 (Sigma-Aldrich, St. Louis, MO, USA, Ref. T8787) in 0.1 M PBS (Sigma-Aldrich, USA, Ref. P4417), pH 7.4. A solution of IB containing 0.5% H_2_O_2_ was used to block the endogenous peroxidase for 15 min at room temperature (RT). The slides with the slices were washed three times with IB and were incubated with Neu-N (Sigma-Aldrich, ref. MAB377 1:100), which is a specific marker for neurons, overnight at 4 °C. The day after, the slides were rinsed three times in IB and then incubated with the secondary antibody (Vector laboratories, ref. BA9200 1:200) for 2 h at room temperature. The sections were then washed three times in IB and then incubated for 90 min at room temperature with the avidin–biotin peroxidase complex (Vectastain ABC kit, Thermo Scientific, ref. #32020, 1:250, Waltham, MA, USA). Each immunohistochemical assay contained slides from each of the areas studied and from each experimental group, and a negative control slide with no primary antibody. The immunostained slides were observed under a light microscope (Zeiss Axioplan Microscope, Oberkochen, Germany) equipped with a high-resolution camera (Zeiss Axioplan 712 color, Germany). The photos were processed using the ZEN3.3 software, version 3.3.89.0000 (Carl Zeiss AG, Oberkochen, Germany). The magnification, light, shine, and contrast conditions were kept constant during the capture process. Figures were prepared using Adobe Photoshop CS4 Extended 10.0 (Adobe Systems, San Jose, CA, USA).

### 2.7. Quantitative Analysis

#### 2.7.1. Retina

To assess the impact of OHT on RGCs and the potential neuroprotective effects of the combination of citicoline and CoQ10, Brn3a+ RGCs and ipRGCs were quantified using a double-blind methodology. Quantification was conducted across all experimental groups.

To quantify Brn3a+ RGCs and ipRGCs, equivalent regions of the retina within the RGC layer were systematically selected and imaged for each retinal whole mount, along both the vertical and horizontal meridians crossing the optic nerve (encompassing superior (dorsal), inferior (ventral), nasal, and temporal zones). Contiguous fields were analyzed to ensure complete coverage of the retinal whole mount, avoiding any omissions or duplications. Each meridian was scanned along its entire length on the x–y axis using the microscope’s motorized stage, resulting in the evaluation of approximately 550 fields in total. Images were acquired at 20x magnification, representing an area of 0.1502 mm^2^ per field [50].

For the quantification of Brn3a+ RGCs, we utilized an automatic MATLAB algorithm developed by our lab for counting Iba-1+ cells. In this application, the minimum distance between RGCs was defined to ensure that each cell was counted only once [56,57]. The counting of melanopsin+ ipRGCs was performed manually, with the number of melanopsin+ ipRGCs being recorded in each field analyzed.

#### 2.7.2. Visual Pathway

NeuN quantification of both the left and right hemispheres of the three visual pathway nuclei (dLGN, SC, and V1) was performed by micro-photographing the immunostained slides at 10x. All the quantifications were performed by two observers blind to the experimental group, and data were used for the statistical analysis only if the mean values had less than 15% inter-observer differences. We performed digital optical densitometry using the ImageJ software, version 1.46r (Research Services Branch-NIH, Bethesda, MD, USA) and the densitometric values are displayed as optical density (O.D.), expressed in arbitrary units.

### 2.8. Statistical Analysis

The statistical analysis was performed using GraphPad Prism 9.0 (GraphPad Prism, La Jolla, CA, USA).

For retinal study statistical significance among the groups vehicle, citicoline + CoQ10, OHT vehicle, contralateral vehicle, OHT–citicoline + CoQ10, and contralateral citicoline + CoQ10 was assessed using the Mann–Whitney U test for unpaired data or the Wilcoxon signed-rank test for comparisons between OHT and contralateral eyes. The following parameters were compared: (i) IOP; (ii) the number of Brn3a+ RGCs; (iii) the number of melanopsin+ ipRGCs. A *p*-value of <0.05 was considered statistically significant.

The relationship between the maximum value of IOP and the retinal neurons (Brn3a+ RGCs and ipRGCs) following a Gaussian distribution was assessed using Pearson’s correlation coefficient (r). Scatter plots illustrate the null hypothesis for pairwise Pearson’s r, with unadjusted *p* values, showing the direction and magnitude of the linear association between the variables.

For the visual pathway study, the statistical significance among the groups vehicle, citicoline + CoQ10, OHT dLGN right, contralateral dLGN left, OHT–citicoline + CoQ10dLGN right, and contralateral citicoline + CoQ10dLGN left was assessed using the Mann–Whitney U test for unpaired data or the Wilcoxon signed-rank test for comparisons between left and right hemispheres. The following parameters were compared: (i) expression of Neu-N+ cells. A *p*-value of <0.05 was considered statistically significant.

## 3. Results

### 3.1. Intraocular Pressure Is Moderate at 24 Hours in the Citicoline + CoQ10 Group

In this study, we compared IOP across different groups of eyes: vehicle, citicoline + CoQ10, OHT, contralateral, OHT–citicoline + CoQ10, contralateral–citicoline + CoQ10. IOP was analyzed at various time points: 24 h, 48 h, 3 days, 5 days, and 7 days.

**Figure 2:**
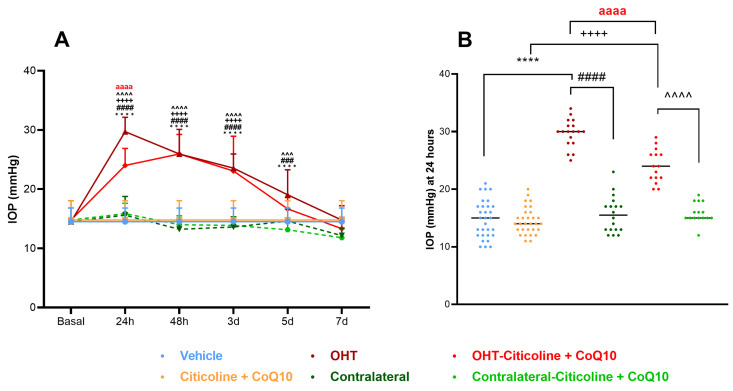
Intraocular pressure (IOP) graphics. (**A**) IOP in different study groups after induction of hypertension. (**B**) IOP data in the different study groups 24 h after induction. Data are mean ± s.e.m.; each data point denotes an individual measure of the IOP. Abbreviations: ocular hypertension (OHT); coenzyme Q10 (CoQ10). Statistical significance indicators: **** *p* < 0.0001 vehicle vs. OHT; ++++ *p* < 0.0001 citicoline + CoQ10 vs. OHT–citicoline + CoQ10; ### *p* < 0.001, #### *p* < 0.0001 OHT vs. contralateral; ^^^ *p* < 0.001, ^^^^ *p* < 0.0001 OHT–citicoline + CoQ10 vs. contralateral–citicoline + CoQ10; aaaa *p* < 0.0001 for OHT vs. OHT–citicoline + CoQ10.

At 24 h, OHT eyes showed a significant increase in IOP compared to all other groups (*p* < 0.0001). At 48 h and 3 days, OHT eyes exhibited a significant increase in IOP with respect to all groups except OHT–citicoline + CoQ10 (*p* < 0.0001). At 5 days, OHT eyes showed a significant increase in IOP compared to contralateral–citicoline + CoQ10 and contralateral (*p* < 0.001), and vehicle (*p* < 0.0001), but not with OHT–citicoline + CoQ10. At 7 days, no significant differences were observed between the groups. These findings suggest that in OHT and OHT–citicoline + CoQ10 eyes IOP remains elevated compared to other groups up to 3 days, then begins to decrease at 5 days, reaching baseline levels at 7 days (Figure 2A).

Additionally, at 24 h, OHT–citicoline + CoQ10 eyes showed a significant increase in IOP compared to citicoline + CoQ10 (*p* < 0.0001). However, OHT–citicoline + CoQ10 eyes showed a significant decrease in IOP compared to OHT (*p* < 0.0001). This suggests that the compound exerts an ocular hypotensive effect only at this time point (Figure 2B).

### 3.2. Retinal Ganglion Cells and Intrinsically Photosensitive Retinal Ganglion Cells

#### 3.2.1. The Combination of Citicoline and CoQ10 Did Not Produce Changes in Retinal Ganglion Cells in Control Eyes

**Figure 3:**
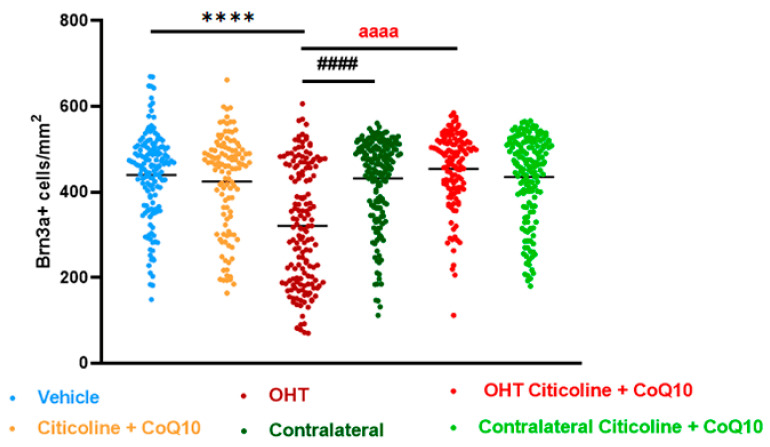
Comparison of total number of Brn3a+ retinal ganglion cells (RGCs) in the different study groups treated with the combination of citicoline and CoQ10 or with vehicle 7 days after OHT induction. Data are mean ± s.e.m.; each data point denotes an individual measure of the Brn3a + RGCs per area of 0.1502 mm^2^. Abbreviations: ocular hypertension (OHT); coenzyme Q10 (CoQ10). Statistical significance indicators: **** *p* < 0.0001 vehicle vs. OHT; #### *p* < 0.0001 OHT vs. contralateral; aaaa *p* < 0.0001 for OHT vs. OHT–citicoline + CoQ10.

Before studying the effects of the combination of citicoline and CoQ10 on the number of RGCs and ipRGCs after OHT laser induction, we first compared vehicle and citicoline + CoQ10 eyes by counting the number of these cells. The results did not show significant differences between the two groups (Figure 3).

#### 3.2.2. The Combination of Citicoline and CoQ10 Protected Retinal Ganglion Cells Against Damage Produced by Laser-Induced Ocular Hypertension

**Figure 4:**
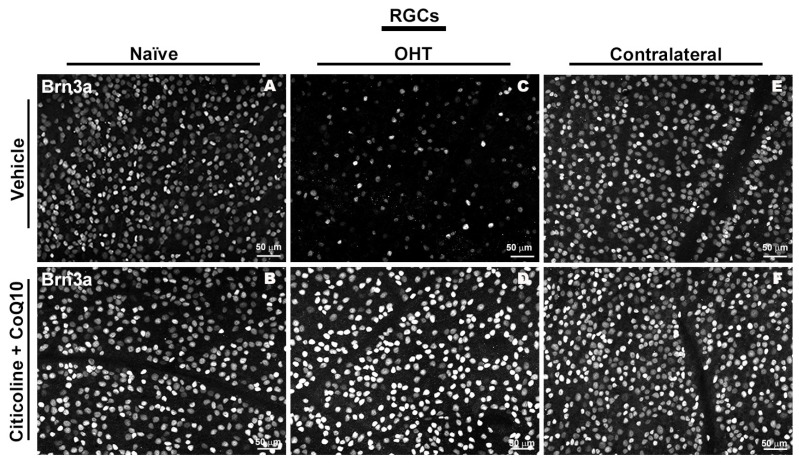
Immunohistochemical images of anti-Brn3a-stained retinal whole mounts 7 days after OHT induction. (**A**) Vehicle, (**B**) citicoline + CoQ10, (**C**) OHT, (**D**) OHT–citicoline + CoQ10, (**E**) contralateral, (**F**) contralateral–citicoline + CoQ10. Abbreviations: ocular hypertension (OHT); coenzyme Q10 (CoQ10).

In the entire retina, OHT eyes showed a significant reduction in the number of Brn3a+ RGCs compared to vehicle and contralateral (*p* < 0.0001). However, citicoline + CoQ10 treatment counteracted this reduction, showing a significant decrease in this cell loss in OHT–citicoline + CoQ10 eyes compared with OHT eyes (*p* < 0.0001), and with no significant differences observed compared with vehicle, contralateral, and contralateral–citicoline + CoQ10 eyes (Figure 3 and Figure 4).

**Figure 5:**
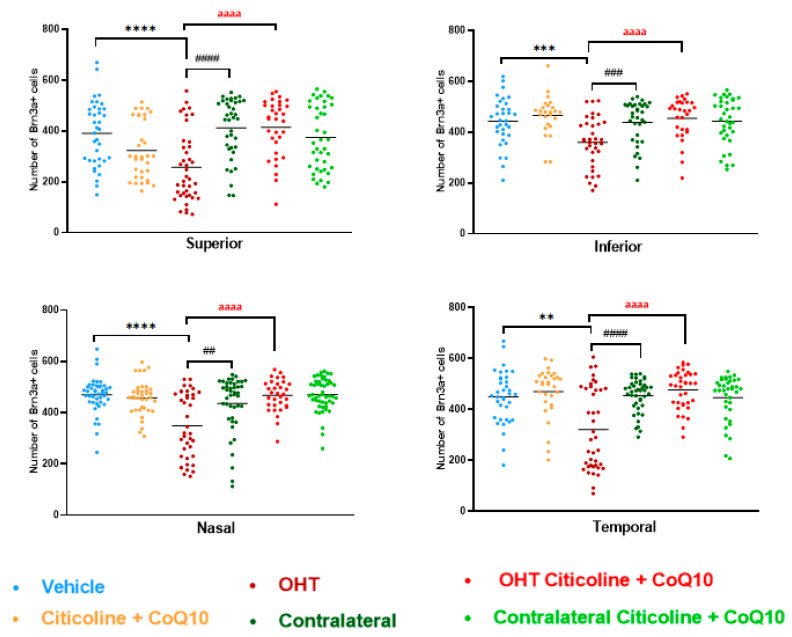
Comparison of number of Brn3a+ retinal ganglion cells (RGCs) in the four retinal sectors, superior, inferior, nasal, and temporal, in the different study groups 7 days after OHT induction. Data are mean ± s.e.m.; each data point denotes an individual measure of the Brn3a + RGCs per area of 0.1502 mm^2^. Abbreviations: ocular hypertension group (OHT); coenzyme Q10 (CoQ10). Statistical significance indicators: ** *p* < 0.01, *** *p* < 0.001, **** *p* < 0.0001 vehicle vs. OHT; ## *p* < 0.01, ### *p* < 0.001, #### *p* < 0.0001 OHT vs. contralateral; aaaa *p* < 0.0001 OHT vs. OHT–citicoline + CoQ10.

In the sectoral analysis of the retina, OHT eyes exhibited a significant reduction in the number of RGCs compared to vehicle and contralateral in the superior (*p* < 0.0001 in all cases), inferior (*p* < 0.001 in all cases), nasal (*p* < 0.0001 in OHT vs. vehicle; *p* < 0.01 in OHT vs. contralateral), and temporal (*p* < 0.01 in OHT vs. vehicle; *p* < 0.0001 in OHT vs. contralateral) sectors (Figure 5). In OHT eyes treated with the combination of citicoline + CoQ10, no loss of RGCs was observed in any of the analyzed sectors, and there were no significant differences compared to the vehicle (Figure 5).

**Figure 6:**
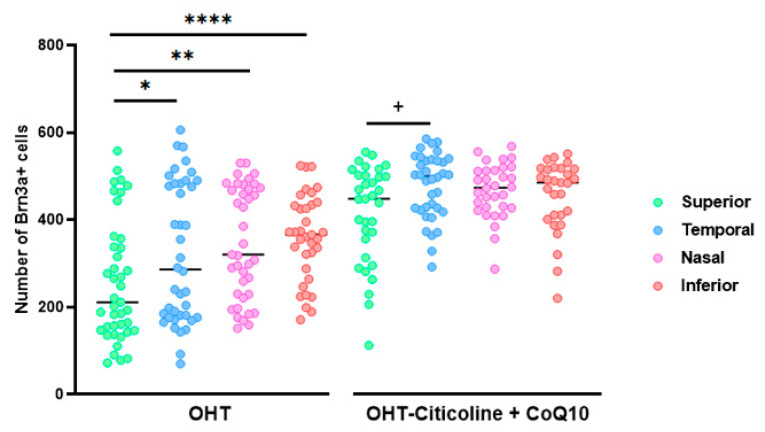
Comparison of the number of Brn3a+ retinal ganglion cells (RGCs) in untreated and citicoline and CoQ10-treated hypertensive eyes in the four retinal sectors, superior, temporal, nasal and inferior, 7 days after OHT induction. Data are mean ± s.e.m.; each data point denotes an individual measure of the Brn3a + RGCs per area of 0.1502 mm^2^. Abbreviations: ocular hypertension (OHT); coenzyme Q10 (CoQ10). Statistical significance indicators: In OHT group: * *p* < 0.05 for superior vs. temporal, ** *p* < 0.01 superior vs. nasal, **** *p* < 0.0001 superior vs. inferior. In OHT–citicoline + CoQ10 group: + *p* < 0.05 superior vs. temporal.

When comparing the number of Brn3a+ RGCs in different sectors of OHT and OHT–citicoline + CoQ10 eyes, OHT eyes showed a significant decrease in the number of cells in the superior sector compared to the temporal (*p* < 0.05), nasal (*p* < 0.01), and inferior (*p* < 0.0001) sectors, indicating that the greatest loss of RGCs occurred in the superior sector. However, in OHT–citicoline + CoQ10 eyes, there was only a slight decrease in the number of RGCs in the superior sector compared to the temporal sector (*p* < 0.05), indicating that in these eyes, there were practically no differences in the number of RGCs between the different retinal sectors (Figure 6).

**Figure 7:**
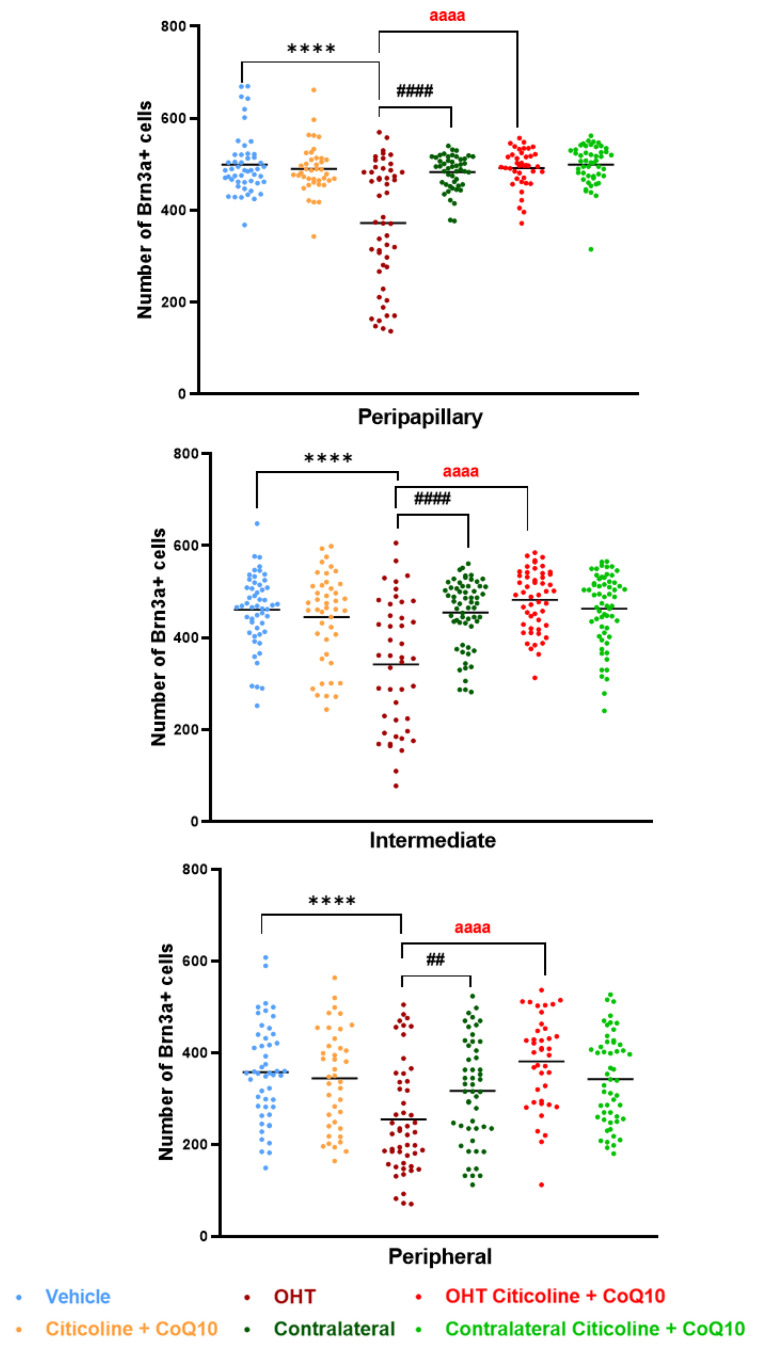
Comparison of number of Brn3a+ retinal ganglion cells (RGCs) in the three retinal areas, peripapillary, intermediate, and peripheral, in the different study groups 7 days after OHT induction. Data are mean ± s.e.m.; each data point denotes an individual measure of the Brn3a + RGCs per area of 0.1502 mm^2^. Abbreviations: ocular hypertension group (OHT); coenzyme Q10 (CoQ10). Statistical significance indicators: **** *p* < 0.0001 vehicle vs. OHT; ## *p* < 0.01, #### *p* < 0.0001 OHT vs. contralateral; aaaa *p* < 0.0001 OHT vs. OHT–citicoline + CoQ10.

When the retina was analyzed by area (peripapillary, intermediate, and peripheral), the OHT eyes showed a significant reduction in the number of Brn3a+ RGCs compared to the vehicle and contralateral eyes in the peripapillary (*p* < 0.0001 in both cases), intermediate (*p* < 0.0001 in both cases), and peripheral areas (*p* < 0.0001 in OHT vs. vehicle; *p* < 0.01 in OHT vs. contralateral). Additionally, the OHT eyes also showed a significant reduction in the number of RGCs when compared to the OHT eyes treated with the combination (citicoline + CoQ10) in all areas (*p* < 0.0001). However, no significant differences were observed when comparing the OHT eyes treated with citicoline + CoQ10 to the vehicle in any of the analyzed areas (Figure 7).

**Figure 8:**
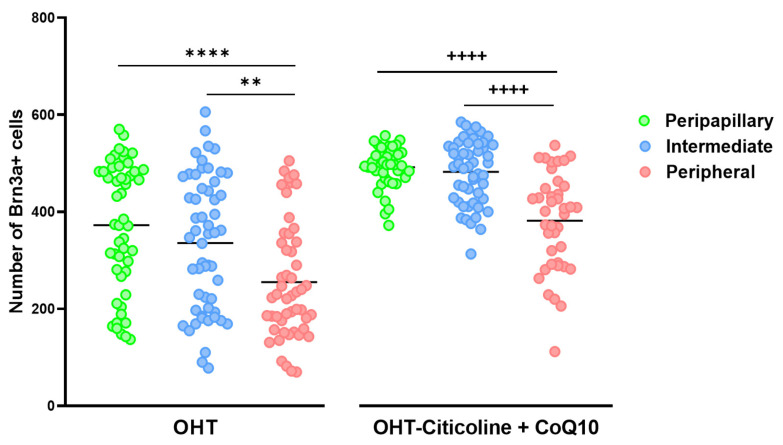
Comparison of the number of Brn3a+ retinal ganglion cells (RGCs) in untreated and citicoline and CoQ10-treated hypertensive eyes in the three retinal areas: peripapillary, intermediate, and peripheral, 7 days after OHT induction. Data are mean ± s.e.m.; each data point denotes an individual measure of the Brn3a + RGCs per area of 0.1502 mm^2^. Abbreviations: ocular hypertension (OHT); coenzyme Q10 (CoQ10). Statistical significance indicators: In OHT group: ** *p* < 0.01 peripheral vs. peripapillary; **** *p* < 0.0001 peripheral vs. intermediate. In OHT–citicoline + CoQ10 group: ++++ *p* < 0.0001 peripheral vs. peripapillary; ++++ *p* < 0.0001 peripheral vs. intermediate.

When comparing the number of Brn3a+ cells in different zones (peripapillary, intermediate, and peripheral) in OHT eyes, a decrease was observed in the peripheral zone compared to the peripapillary zone (*p* < 0.0001) and the intermediate zone (*p* < 0.01). This distribution is also observed in OHT eyes treated with citicoline + CoQ10 (peripheral vs. peripapillary and peripheral vs. intermediate; *p* < 0.0001 in both cases). The difference between untreated and treated OHT eyes is that the number of Brn3a+ RGCs is higher in all zones in animals treated with citicoline + CoQ10 due to the prevention of RGC loss (Figure 8).

#### 3.2.3. Ocular Hypertension Does Not Affect the Number of Intrinsically Photosensitive Retinal Ganglion Cells (ipRGCs) That Express Melanopsin in Either Untreated Ocular Hypertensive Eyes or Those Treated with the Combination of Citicoline and CoQ10

**Figure 9:**
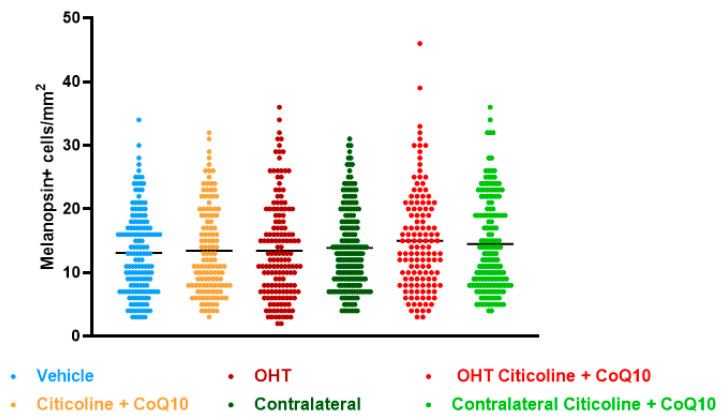
Comparison of total number of melanopsin+ intrinsically photosensitive retinal ganglion cells (ipRGCs) in the different study groups treated with the combination of citicoline and CoQ10 or with vehicle 7 days after OHT induction. Data are mean ± s.e.m.; each data point denotes an individual measure of the melanopsin+ ipRGCs per area of 0.1502 mm^2^. Abbreviations: ocular hypertension (OHT); coenzyme Q10 (CoQ10).

**Figure 10:**
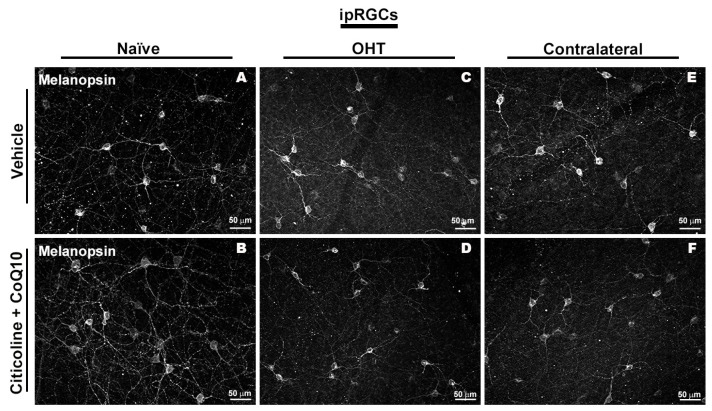
Immunohistochemical images of anti-melanopsin-stained retinal whole mounts 7 days after ocular hypertension induction. (**A**) Vehicle, (**B**) citicoline + CoQ10, (**C**) OHT, (**D**) OHT–citicoline + CoQ10, (**E**) contralateral, (**F**) contralateral–citicoline + CoQ10. Abbreviations: ocular hypertension (OHT); coenzyme Q10 (CoQ10).

**Figure 11:**
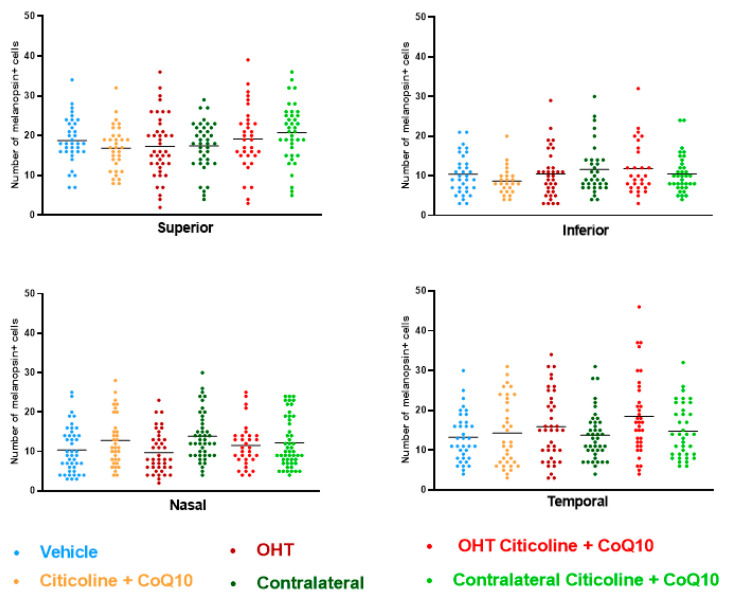
Comparison of number of melanopsin+ intrinsically photosensitive retinal ganglion cells (ipRGCs) in the four retinal sectors, superior, inferior, nasal, and temporal, in the different study groups 7 days after OHT induction. Data are mean ± s.e.m.; each data point denotes an individual measure of the melanopsin+ ipRGCs per area of 0.1502 mm^2^. Abbreviations: ocular hypertension (OHT); coenzyme Q10 (CoQ10).

**Figure 12:**
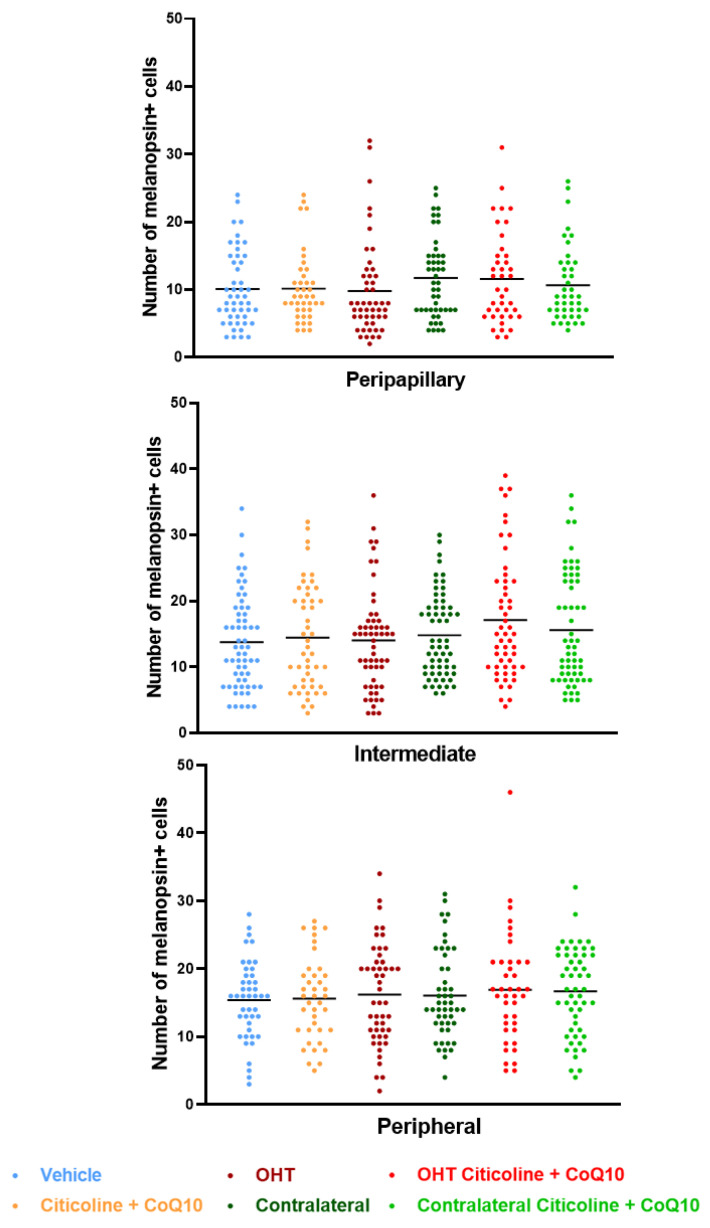
Comparison of total number of melanopsin+ intrinsically photosensitive retinal ganglion cells (ipRGCs) in the three retinal areas, peripapillary, intermediate, and peripheral, in the different study groups 7 days after OHT induction. Data are mean ± s.e.m.; each data point denotes an individual measure of the melanopsin+ ipRGCs per area of 0.1502 mm^2^. Abbreviations: ocular hypertension (OHT); coenzyme Q10 (CoQ10).

Regarding the number of melanopsin+ ipRGCs, no significant differences were found between the different groups (vehicle, citicoline + CoQ10, OHT, OHT–citicoline + CoQ10, contralateral, and contralateral–citicoline + CoQ10) in either the total retina (Figure 9 and Figure 10), the sectoral analysis (Figure 11), or the area analysis (Figure 12). This indicates that in OHT and OHT–citicoline + CoQ10 eyes there was no loss of melanopsin+ ipRGCs.

### 3.3. IOP Has No Correlation in the Loss of RGCs in the Citicoline + CoQ10-Treated Eyes

The correlation between the maximum value of IOP and the number of RGCs was analyzed in the untreated groups and the citicoline + CoQ10-treated groups.

**Figure 13:**
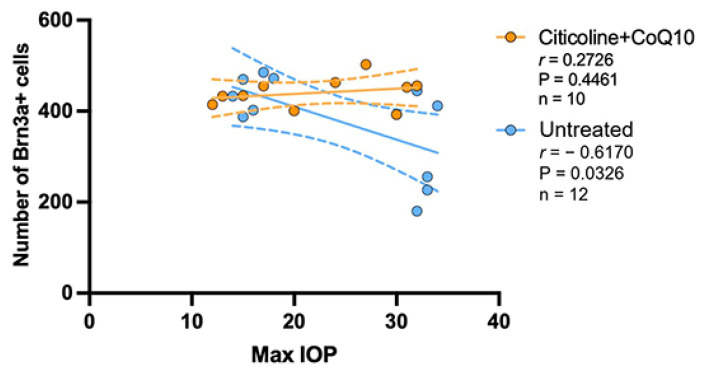
Pearson’s correlation coefficient (r) analysis between the number of Brn3a+ RGCs and maximum IOP in the untreated eyes (vehicle and OHT–vehicle groups) and the treated eyes (citicoline + CoQ10 and OHT–citicoline + CoQ10).

The IOP value has a negative strong correlation with the loss of RGCs in the untreated group (r = −0.6170, *p* <0.05); however, no correlation was found in the citicoline + CoQ10-treated eyes (r = 0.2726, *p* = 0.4461) (Figure 13).

**Figure 14:**
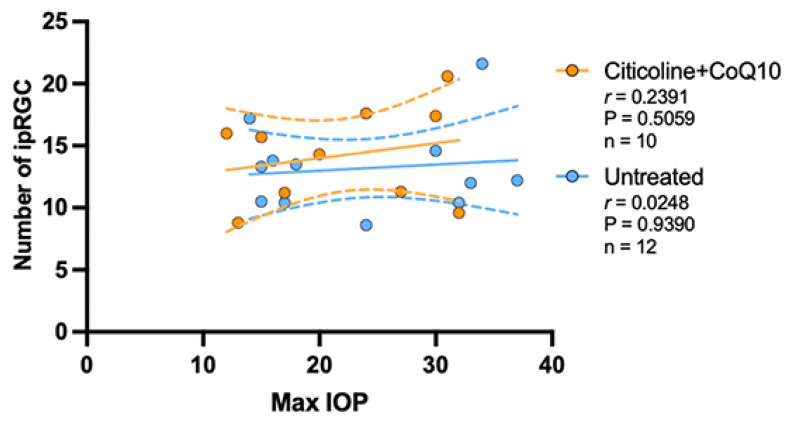
Pearson’s correlation coefficient (r) analysis between the number of melanopsin+ ipRGC and maximum IOP in the untreated eyes (vehicle and OHT–vehicle groups) and the treated eyes (citicoline + CoQ10 and OHT–citicoline + CoQ10).

The analysis of the correlation between the maximum value of IOP and the number of ipRGCs revealed no correlation between the loss of ipRGCs and IOP values, either in untreated eyes (r = 0.0248, *p* > 0.05) or in those treated with citicoline + CoQ10 (r = 0.2391, *p* > 0.05) (Figure 14).

### 3.4. The Combination of Citicoline and CoQ10 Protected the Dorsolateral Geniculate Nucleus (dLGN) Against Damage Produced by Laser-Induced Ocular Hypertension

In this study, we compared NeuN expression in three main visual pathway nuclei, dorsolateral geniculate nucleus (dLGN), superior colliculus (SC), and visual cortex (V1), at 7 days post-surgery.

**Figure 15:**
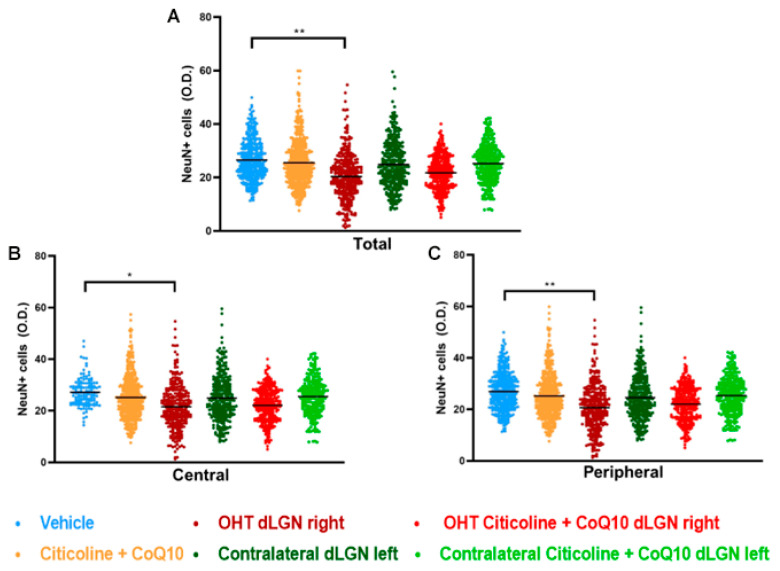
Analysis of NeuN+ cells (OD) of the dorsolateral geniculate nucleus in the total (**A**), central zone (**B**), and peripheral zone (**C**) in the different study groups 7 days after ocular hypertension induction. Data are mean ± s.e.m.; each data point denotes an individual measure of the NeuN+ cells (OD). Abbreviations: ocular hypertension group (OHT); coenzyme Q10 (CoQ10). Statistical significance indicators: * *p* < 0.05, ** *p* < 0.01 for vehicle vs. OHT dLGN right.

**Figure 16:**
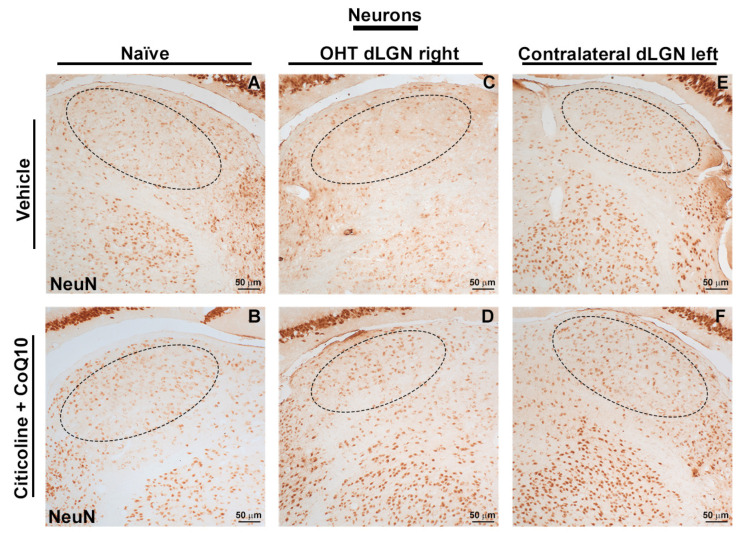
Immunohistochemical images of brain sections of the dorsolateral geniculate nucleus stained with anti-NeuN 7 days after induction of ocular hypertension. (**A**) Vehicle, (**B**) citicoline + CoQ10, (**C**) OHT dLGN right, (**D**) OHT dLGN right–citicoline + CoQ10, (**E**) contralateral dLGN left, (**F**) contralateral dLGN left–citicoline + CoQ10. Abbreviations: ocular hypertension (OHT); coenzyme Q10 (CoQ10).

When analyzing the total expression of NeuN in the dLGN (Figure 15A and Figure 16), the OHT dLGN right showed a significant decrease in NeuN expression compared to the vehicle group (*p* < 0.01). Similar results were obtained when analyzing the central zone (Figure 15B) of the dLGN (*p* < 0.05) and the peripheral zone (Figure 15C) of the dLGN (*p* < 0.01), although this effect was milder in the latter. In eyes treated with the combination of citicoline + CoQ10, no decrease in NeuN expression was observed, either in the total analysis (Figure 15A) or in the sectoral analysis (Figure 15B,C), with no significant differences compared to the vehicle group.

**Figure 17:**
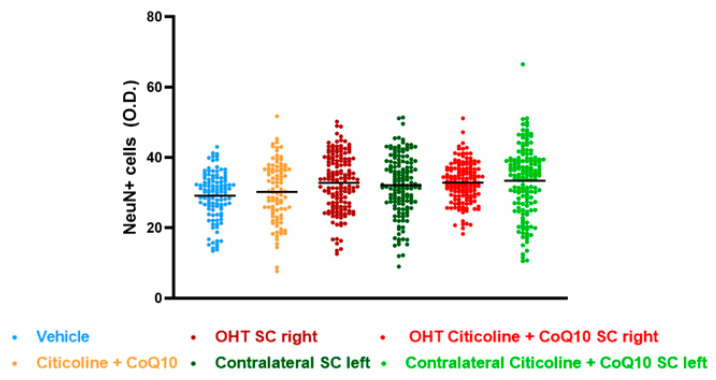
Analysis of NeuN+ cells (OD) of the superior colliculus (SC) in the different study groups treated with the combination of citicoline and CoQ10 or with vehicle 7 days after OHT induction. Data are mean ± s.e.m.; each data point denotes an individual measurement of the NeuN+ cells (OD). Abbreviations: ocular hypertension (OHT); coenzyme Q10 (CoQ10).

**Figure 18:**
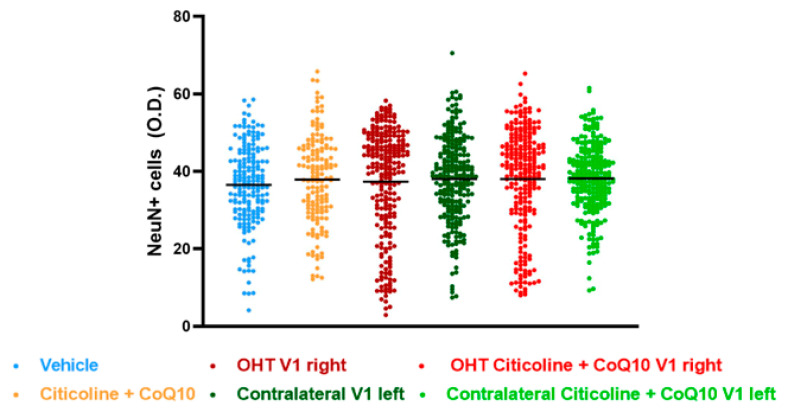
Analysis of NeuN+ cells (OD) of the visual cortex (V1) in the different study groups treated with the combination of citicoline and CoQ10 or with vehicle 7 days after ocular hypertension induction. Data are mean ± s.e.m.; each data point denotes an individual measure of the NeuN+ cells (OD). Abbreviations: ocular hypertension (OHT); coenzyme Q10 (CoQ10).

The analysis of NeuN expression in the other two relay nuclei, SC and V1, showed no significant differences between the experimental groups (Figure 17 and Figure 18).

## 4. Discussion

In this study, we suggest that the combination of citicoline + CoQ10 may exert a neuroprotective effect on RGCs and in the dLGN of the visual pathway in a laser-induced OHT model in mice. This is the first study to demonstrate this neuroprotective effect in a glaucoma model, as only one previous study has analyzed the beneficial effect of this combination in rat astrocyte cultures exposed to oxidative stress [58].

We utilized a unilateral laser-induced hypertension model, which causes a transient elevation of IOP that triggers significant and sectorial death of RGCs [51,59,60]. This model is useful for analyzing how IOP can damage the retina and for evaluating neuroprotective substances [50,52,59].

In our study, the OHT eyes showed a significant increase in IOP on the first day post-photocoagulation. However, in OHT eyes treated with citicoline + CoQ10, this increase in IOP was less pronounced, suggesting that the compound attenuated the IOP increase at this early time.

Previous studies have not found reductions in IOP following the administration of citicoline or CoQ10 separately in various glaucoma models [41,43,47,48,61,62]. Additionally, no changes in IOP were observed in glaucoma patients treated with citicoline [49,63,64,65,66,67,68,69,70,71,72,73,74,75] or CoQ10 + vitamin E [76].

The smaller rise in IOP in OHT animals treated with citicoline and CoQ10 at 24 h post-laser could be due to the combined effects of both drugs. At 24 h post-laser, there is a greater elevation of IOP and an inflammatory process [55]. In rat astrocyte cultures exposed to OS, the combination of citicoline and CoQ10 reduced the gene expression of pro-apoptotic and inflammatory proteins (BAX, BCL-2, SOD2, CRLS1, NFkβ, TNFα) more than when used separately [58]. This suggests that the combination may better protect against the early inflammatory process, resulting in less elevated IOP peaks.

To evaluate the potential neuroprotective effect of the citicoline + CoQ10 combination, we selected the 7 days following the induction of OHT, because previous studies have shown that significant RGC loss (33%) occurs within this timeframe [24,50,51,59,60]. We observed a significant decrease in the number of Brn3a+ RGCs in OHT eyes at this time point, both in the entire retina and in different retinal sectors (superior, inferior, nasal, and temporal) and retinal areas (peripapillary, intermediate, and peripheral). The most prominent loss was observed in the superior region and peripheral area. However, in OHT eyes treated with citicoline + CoQ10, this RGC loss did not occur, suggesting that this compound can protect against RGC death even in eyes that have experienced higher levels of IOP.

The melanopsin + ipRGCs, showed no changes in their number 7 days after the OHT induction, either in the OHT eyes or in the OHT eyes treated with citicoline + CoQ10, demonstrating the resistance of these cells to high IOP levels.

Although this remains a topic of debate, several studies have reported a higher survival rate of ipRGCs compared to RGCs following different protocols of IOP elevation [5,77,78]. The reduced vulnerability of ipRGCs may be attributed to several factors, including their relatively high level of mTOR activity, which is associated with axonal regeneration [79,80]; the augmented trophic support from their axonal collaterals within the eye [81,82]; and possibly, the shorter axonal pathways of M1 ipRGCs, as longer axons tend to suffer more damage [83].

Additionally, 7 days after the induction of OHT we also found a reduction in NeuN+ neurons within the dLGN, both in the peripheral and central regions of the contralateral/right dLGN. Actually, the peripheral zone that receives afferents mostly from the contralateral eye seems to be more affected [84,85,86]. That is, the damaged OHT eye, that presents a significant reduction in Brn3a+ cells, in the temporal and nasal retina among other areas, given the retinotopic organization, projects RGC axons mainly to the superior or inferior portions of the dLGN, respectively [85], that is, the peripheral dLGN. On the other hand, the observed decrease in NeuN expression, also in the central region of the contralateral/right dLGN, might be due to the plausible local neuroinflammatory response to the neuronal damage observed in the periphery or caused by the direct loss of the few RGC afferents from the damaged OHT eye.

Our results are in accordance with previous studies reporting the effects of glaucoma on LGN neurons both in humans [8,87] and primate models [88], and are similar to the glaucoma-induced hypoxia evolution from the retina through the visual pathway already reported, in which damage was first described in the LGN, followed by an effect in the SC [9]. Other studies have reported a decrease in neurons in both relay nuclei, i.e., dLGN and SC, by using a similar animal model in rats [89]. Actually, we may have expected similar damage in the SC since, in rodents, most RGC axons project directly to the SC [8,90]. However, in this study, no alterations in the neuronal population within the SC were reported. The lack of neuronal damage within the SC might be due to the fact that it receives a lot of information from the intrinsically photosensitive GCs (ipGCs) that are resistant to the induced OHT damage [91]; or to the fact that this SC is a multimodal sensorimotor structure, receiving information not only from the visual pathway but from auditory and somatosensory pathways, and additionally integrating information for the initiation of motor patterns [92,93]. The absence of neuronal damage in the SC may not imply a lack of effect in this relay nucleus; an effect could possibly occur over a longer period of time, perhaps due to trans-neuronal degeneration. Moreover, in case of a possible functional alteration in SC, it may also affect the dLGN through collateral branches coming from the CS [94], thus further enhancing the neuronal damage caused by the direct loss of RGCs in the retina. A similar explanation could be applied for V1. To further investigate the damage along the visual pathway, it would be interesting to evaluate and quantify axonal projections from the retina using anterograde tracers.

As for the RGCs in the OHT dLGN, we did not find significant differences in the animals treated with the compound citicoline and CoQ10 compared to the vehicle group, suggesting that this compound could have some effect on the dLGN.

Despite there being no studies available that analyze the combination of citicoline and CoQ10 on RGC survival in glaucoma models, some studies have examined the actions of each drug separately.

On the one hand, citicoline has shown neuroprotective properties in retinal cell cultures by reducing glutamate-induced apoptosis [95], as well as in a rat model of partial optic nerve crush [96]. On the other hand, CoQ10 has shown neuroprotective properties in several glaucoma models. For instance, in DBA/2J mice, CoQ10 promoted RGC survival by 29% [47], while ubiquinol, the reduced form of CoQ10, promoted RGC survival by 50% [62]. Additionally, in an ischemia–reperfusion model, CoQ10 reduced RGC loss by 10.3% in rats [97], and promoted RGC survival by approximately 21% in mice [43]. This effect was also reported for ubiquinol, which promoted RGC survival by 17% in the same model [48].

As seen in these studies, there is greater RGC survival following treatment with either drug alone, but complete neuroprotection is not achieved, as observed at 7 days post-OHT induction with the combined administration of citicoline + CoQ10. This is consistent with other studies showing that drug combinations can promote greater RGC survival in glaucoma models. In the Morrison model of glaucoma induced by hypertonic saline in episcleral veins in rats, treatment with CoQ10 + α-tocopherol provided almost complete protection of RGCs in OHT retinas [41]. Furthermore, in the same model, intravitreal treatment with microspheres loaded with dexamethasone, melatonin, and CoQ10 in a single formulation inhibited RGC degeneration associated with OHT [98].

Therefore, the combination of citicoline and CoQ10 used in our study may have synergistic effects, potentially acting on multiple neuroprotective pathways, providing a more potent therapeutic strategy to reduce neurodegeneration in neuronal diseases such as glaucoma.

There are several mechanisms suggesting that CoQ10 can protect RGCs against mitochondrial dysfunction and cell death under conditions of ischemia and elevated intraocular pressure, such as in glaucoma. CoQ10 modulates apoptosis by decreasing Bax and increasing pBad, which activates Bcl-xL, helping to maintain mitochondrial homeostasis and prevent apoptosis survival [43,47,48,62]. CoQ10 also preserves mitochondrial DNA and the expression of the complex IV protein TFAM/OXPHOS, protecting against glutamate excitotoxicity and oxidative stress [47,62]. Ubiquinol, the reduced form of CoQ10, enhances the expression of OXPHOS complex II, increasing mitochondrial respiratory capacity [62]. Additionally, CoQ10 improves glutamate transporter function, reducing extracellular glutamate and preventing RGC death [97,99], while also reducing oxidative stress by enhancing the PKB/Akt pathway [99].

Citicoline has shown various benefits in glaucoma patients, including improvements in visual acuity, visual field, electroretinogram, visual evoked potentials, and structural techniques such as optical coherence tomography (OCT) [37,49,63,64,65,66,67,68,69,70,71,72,73,74,75]. Citicoline may stimulate phospholipid synthesis, aiding in the repair and regeneration of damaged neuronal membranes [27] and enhancing the function of key elements in the visual pathway [72]. It reduces glutamate concentration by increasing glutamate transporter expression in astrocytes [38] and counteracts neuronal damage in retinal cultures treated with glutamate [95]. In a rat model of retinal damage induced by kainic acid, citicoline treatment showed less pronounced retinal thinning [100]. Therefore, citicoline could mitigate glutamate-induced excitotoxic damage, promoting neuroprotection in glaucoma.

The significant neuroprotective effect reported here for the citicoline and CoQ10 combination could also be attributed to the administration protocol employed, that was initiated before the OHT-induced damage and continued until the end of the study. If this administration protocol were translated to clinical practice, that is, if it were administered to patients with OHT or in the early stages of glaucoma, it could be effective before the full neurodegenerative process was triggered.

However, the study has several limitations: the evaluation of the drugs in combination and not separately, although the literature shows that, in animal models, these drugs can only reduce RGC loss, but not completely prevent it; the duration of the study, limited to 7 days—longer periods should be tested to further demonstrate the efficacy of the citicoline and CoQ10 combination; last, but not least, the effectiveness of this combination should also be demonstrated in female animals, filling the gender gap still present in glaucoma research.

## 5. Conclusions

This study demonstrates that the oral administration of the citicoline and CoQ10 combination can exert a neuroprotective effect against RGC death and visual pathway alterations in an established rodent model of OHT. It also provides evidence that neuroprotective therapies should be applied before significant anatomical or functional loss occurs to achieve maximum effectiveness. Consequently, neuroprotective therapies used adjunctively with hypotensive drugs could be fundamental in the treatment of glaucoma.

## Data Availability

The data presented in this study are available on request from the corresponding author. The data are not publicly available due to patentability concerns.
